# Effect of the Darolac^®^ (Oralis SB^®^) Probiotic Formulation on Oral Health: A Narrative Review

**DOI:** 10.3390/microorganisms13020408

**Published:** 2025-02-13

**Authors:** Lya Blais, Noémie Auclair-Ouellet, Annie Tremblay, Sylvie Binda

**Affiliations:** Rosell Institute for Microbiome and Probiotics, 6100 Royalmount Avenue, Montreal, QC H4P 2R2, Canada; lblais@lallemand.com (L.B.); nauclairouellet@lallemand.com (N.A.-O.); atremblay@lallemand.com (A.T.)

**Keywords:** probiotics, periodontal conditions, gingivitis, periodontitis

## Abstract

Gingivitis and periodontitis are prevalent periodontal conditions associated with dysbiosis of the oral cavity, which leads to inflammation and bleeding of gums, loss of tooth attachment, and degradation of the underlying bone structure. The standard adjunctive treatment for periodontal conditions, chlorhexidine mouthwash, is effective but is associated with several side effects. Probiotics have been explored as an alternative solution that promotes oral health by restoring homeostasis in the oral cavity. This review presents a summary of clinical trials using the Darolac^®^ (Oralis SB^®^) probiotic formulation (*Lactobacillus acidophilus* Rosell^®^-52, *Lactobacillus rhamnosus* Rosell^®^-11, *Bifidobacterium longum* Rosell^®^-175 and *Saccharomyces boulardii* CNCM I-1079) as a mouthwash to support the maintenance of oral health or the restoration of its balance. In reviewed studies, Darolac^®^ is compared to a placebo or other common solutions for periodontal conditions, including chlorhexidine mouthwash. Studies show that Darolac^®^ is as effective or even superior to other available solutions, which supports its use as an effective adjuvant to oral health. The effects of Darolac^®^ on the reduction in oral pathogens and markers of oral dysbiosis are reviewed, and the association between periodontitis, inflammation, and systemic diseases, as well as their implications and the use of probiotics in the periodontal field, are discussed.

## 1. Introduction

The oral microbiota is a complex community of bacteria, viruses, archaea and fungi that interact and form a unique biofilm [1,2]. Oral biofilm is found all around the surface of the oral cavity and constitutes what is known as plaque on the surface of teeth [1,3]. Like other microbiological niches, the oral microbiota can be in a state of imbalance, or dysbiosis. Several behaviors have an important impact on the oral microbiota, including diet, smoking, drug use, and oral hygiene practices [4,5]. All these behaviors can contribute to dysbiosis and colonization of the oral cavity by opportunistic pathogens, which may lead to diseases [6].

Oral health conditions affect around 3.5B people in the world, across developed and developing countries [5,7]. The most common include periodontal conditions (e.g., periodontitis, gingivitis), tooth caries, and oral cancer. The prevalence of periodontal conditions varies across countries but is high in general. According to data compiled by the World Health Organization (WHO), in 2019, the prevalence of severe periodontal conditions in individuals aged 15 years and older was 24.7% in Canada, 17.5% in China, 21.8% in India, and 15.7% in the United States of America [8,9,10,11]. It is worth noting that these estimates represent the percentage of individuals who have severe periodontal conditions and do not account for individuals affected by less severe forms. For instance, a meta-analysis by Janakiram et al. [12] concluded that the overall prevalence of periodontitis in India was 51%. A study based on the National Oral Health Survey in China that divided results according to three age groups found that the overall prevalence of periodontitis was 52.8% in adults aged 35–44, 69.3% in adults aged 55–64, and 64.6% in adults aged 65 to 74 [13]. In comparison, the prevalence of severe periodontitis was 10.6%, 37.3%, and 43.5% in the three age groups, respectively, which highlights the effect of age on both prevalence and severity. Furthermore, the Global Burden of Diseases, Injuries, and Risk Factors Study [14] compiled years lost to disability for 354 diseases and injuries across 195 countries and reported that from 1990 to 2017, oral conditions (mainly periodontitis and caries) contributed the most years lost to disability in age-standardized prevalence rates [14]. Some countries, including India, China, the United States of America, Pakistan and Indonesia, are known to have a high disease burden related to periodontal conditions, caries and oral cancer [15].

Periodontal conditions affect the gingiva (gums) and the underlying bone structure [16]. Common periodontal conditions include gingivitis and periodontitis. Gingivitis is defined as inflammation of gum tissues characterized by redness, swelling and bleeding of gums [17,18]. It is a reversible inflammatory state that is initiated by plaque build-up on the teeth. As dental plaque accumulates, the oral cavity is colonized by a greater load of pathogenic and opportunistic bacteria, resulting in inflammation in the gingiva [19]. Gingivitis is associated with a shift in the microbial population of the subgingival plaque which forms a new niche that is favorable to anaerobic bacteria [20]. Some taxa have been associated with gingivitis, namely *Streptococcus* spp., *Veillonella* spp., *Prevotella* spp., *Lautropia* spp., *Haemophilus* spp., *Leptotrichia* spp., *Selenomonas* spp. and TM7 [20,21]. However, gingivitis may be caused by risk factors other than bacterial infection, including hormonal changes during puberty and pregnancy, drug use, and smoking [19,22]. If it is not managed properly, it may lead to a more severe periodontal condition: periodontitis [23].

Periodontitis is characterized by inflammation, bone loss and infection that, if not managed properly, can result in pain, loss of tooth attachment, and severe gum swelling, causing the formation of pockets between the teeth and gums where bacteria can grow [23,24,25]. As is the case for gingivitis, many habits can contribute to the occurrence of periodontitis, such as smoking and poor oral hygiene [4,5]. The prevalence of periodontitis is related to a general decline in oral health that occurs with age [12]. Some diseases, such as diabetes and obesity, as well as hormonal changes related to pregnancy, menopause and stress, have also been associated with the development of periodontitis [5,26,27].

Although its etiology is still under debate, the occurrence of periodontitis is associated with a shift in the oral microbiota composition, as well as an increase in microbial load [28]. The accumulation of dental plaque in periodontitis leads to the proliferation of pathogenic bacteria within this biofilm, which causes inflammation [29]. The first pathogens identified as being involved in periodontitis were *Porphyromonas gingivalis*, *Tannerella forsythia* and *Treponema denticola,* known as “red complex” bacteria [30,31,32]. The model of oral bacteria as a complex divides oral bacteria into three groups: commensal bacteria of the oral cavity (green complex), bridging bacteria that associate with both commensals and pathogens (orange complex), and oral pathogens (red complex) [33]. Over the years, other pathogens have been associated with periodontitis, notably from the genera *Porphyromonas*, *Synergistetes*, *Peptostreptococcaceae*, *Actinomyces* and *Filifactor* [6].

As mentioned, gingivitis is a reversible condition that is easily prevented and treated by good oral hygiene practices. Since gingivitis is caused by an accumulation of plaque, its removal by a professional will help reduce inflammation [19,34,35]. If the condition is more severe, antiseptic mouthwashes that contain chlorhexidine (CHX) may be prescribed, along with scaling and root planing by a professional [19]. The management of periodontitis may involve behavioral changes, such as smoking cessation, since these behaviors play an important role in this condition [4,5]. While CHX mouthwash remains a common adjunctive solution for periodontal conditions, its use is associated with a number of unpleasant side-effects, including tooth discoloration, tongue discoloration, dry mouth, and taste disturbance [36,37,38,39]. Less common side effects include burning or “pins and needles” sensations, desquamation of the oral mucosa, swelling of the parotid gland, and in some rare cases, allergic reactions [36]. Importantly, as a broad-spectrum anti-microbial agent, CHX can limit the growth of opportunistic bacteria and pathogens, but also of commensals of the oral cavity, potentially contributing to dysbiosis [36,39]. Furthermore, reductions in oral bacteria involved in gluten or nitrate metabolism from the *Actinomyces*, *Neisseria*, *Rothia*, and *Veillonella* spp. have been thought to contribute to further negative downstream effects on immune, digestive, and cardiovascular health [39,40,41,42]. Research has been conducted to identify alternatives with equivalent efficacy that avoid these effects. For instance, herbal mouthwashes that contain medicinal plants with anti-inflammatory properties (e.g., turmeric, chamomile, echinacea) have been tested for the management of gingivitis [19,43], and studies have investigated the effects of herbal mouthwashes in combination with mechanical plaque removal to replace antiseptic mouthwashes in the management of periodontitis [44,45]. In general, the public has been seeking natural health products that confer similar benefits without relying on harsh chemicals. Considering that periodontal conditions are related to oral dysbiosis, approaches that promote the restoration of a healthy oral microbiota may represent an especially interesting avenue.

Probiotics, defined as “live microorganisms that, when administered in adequate amounts, confer a health benefit on the host” [46], are an emerging natural alternative for the maintenance of various health-related aspects. Several probiotic formulations are available for oral health as adjunctive solutions to support the management and reduce risks of conditions like gingivitis and periodontitis. Homayouni Rad et al. [47] recently published a comprehensive review on the use of probiotics and postbiotics in oral health, where they grouped probiotics by genus (*Bifidobacteria*, *Lactobacilli*), and discussed their effects on pathogens and oral health conditions. However, with major advances accomplished in the characterization of probiotic strains in recent years, there is increasing consensus that the effects of probiotics are not generalizable across a given genus or species, and that they are in fact specific to bacterial strains and to the synergistic effects of combinations of specific strains [48], which is also acknowledged by Homayouni Rad et al. [47]. This narrative review presents a summary of findings from clinical trials using Darolac^®^, a probiotic formulation distributed by Aristo Pharmaceuticals in India, a country where around 51% of the population is affected by periodontal conditions according to a recent meta-analysis [12]. The Darolac^®^ formulation is also commercialized under the name Oralis SB^®^ in several countries. It contains four probiotic strains, *Lactobacillus acidophilus* Rosell^®^-52, *Lactobacillus rhamnosus* Rosell^®^-11, *Bifidobacterium longum* Rosell^®^-175 and *Saccharomyces boulardii* CNCM I-1079, for a total of 2.5 billion colony forming units (CFU) per 2 g manufactured sachet [49]. Rosell^®^-52 is also known as *Lactobacillus helveticus* and Rosell^®^-11 is also known as *Lacticaseibacillus rhamnosus*. Excipients (potato starch and magnesium stearate) complete the formulation. The probiotic strains entering in the composition of Darolac^®^ have been recognized as safe to use by the Natural and Non-Prescription Health Products Directorate (NNHPD) of Canada. Health claims recognized by the NNHPD for this probiotic formulation include that it supports/improves oral health; it supports a healthy oral microbiota/microbiome balance; it promotes gum health; it reduces dental plaque and helps reduce/slow plaque build-up; and it helps support healthy *Candida* levels in oral microbiome. Darolac^®^ has been used in several clinical trials focused on oral health and has shown interesting results. This comprehensive review synthesizes results for the use of Darolac^®^ as an adjunct to the restoration and maintenance of oral health in Indian populations.

## 2. Study Characteristics

Table 1 presents study design, total number of participants, population and age range, study arms, Darolac^®^ (Aristo Pharmaceuticals Private Limited, Mumbai, India) regimen, baseline visit details, clinical parameters measured and assessment timepoints, adverse events record and main findings. Six studies were conducted with children [50,51,52,53,54,55] (total age range: 6 to 16) and seven with adults [56,57,58,59,60,61,62] (total age range: 18 to 80; maximum age was not reported in one study [61]). Considering the different aims and clinical indications in those studies, results will be reviewed for those two age groups separately. In general, studies were conducted in participants with no systemic or oral health condition, but one study included participants with gingivitis [57] and two studies included participants with periodontitis [60,61]. Additionally, one study was conducted with adults who had completed radiotherapy for head-and-neck cancer within the previous two weeks [59].

Except for three studies [54,56,62], Darolac^®^ sachets with a dose of 1.25 billion CFU were provided to study participants. Participants were instructed to mix the content of the sachet with water and rinse their mouth with this solution once [50,51], twice [52,53,55,57,58,60,61] or three times [59] daily. Bande et al. [54] provided Darolac^®^ sachets with a dose of 2.5B CFU that were used once daily. Boyapati et al. [62] and Jothika et al. [56] did not specify the probiotic dosage other than that the Darolac^®^ mouthwash was used twice daily.

In seven studies, oral prophylaxis (scaling and root planing, gum debridement) was completed after the collection of baseline measures [51,54,56,58,60,61,62]. In general, participants were instructed to observe strict compliance with the mouthwash regimen and to maintain their normal oral hygiene practices otherwise. In the study of Gedam et al. [55], participants were instructed to use non-fluoridated toothpaste, while in three other studies, participants were provided with a toothbrush and toothpaste to ensure the equivalence of oral hygiene practices across groups [53,58,62].

Participants were asked to use the probiotic or comparator for 14 days in eight studies [50,51,52,54,55,57,58,60], 28 or 30 days in four studies [56,59,61,62] and three months (90 days) in one study [53]. Measures were taken at baseline and at the end of the intervention in all studies. Some studies also collected data during the intervention [56,58,62] and included follow-up measures after discontinuation of product use [51,61].

Two main comparators were used in the clinical trials: CHX mouthwash, a common adjunctive solution for periodontal conditions, and a placebo mouthwash, which corresponded to distilled water or saline solution in most studies. Other comparators included herbal mouthwash, povidone–iodine mouthwash, clotrimazole, and other probiotic formulations.

In terms of outcomes, most studies reported two clinical indicators of oral health: the gingival index (GI) and plaque index (PI) [63,64]. Five studies investigated the presence of oral pathogens in plaque or saliva samples [50,55,56,59,60], and one study reported saliva pH and lgA levels [61]. Lastly, one study compared taste acceptance of different mouthwash solutions in children [55].

## 3. Darolac^®^ for Children

Developing good oral hygiene practices early in life helps maintain oral health and prevent the development of periodontal conditions. However, children may lack the fine motor skills and patience necessary to brush their teeth twice daily and floss as recommended by oral care professionals. This may particularly be the case for children with special needs [53]. The use of products that are convenient and easy to use, such as mouthwashes, has been explored for their role as adjuvants to good oral health.

### 3.1. Clinical Indicators of Oral Health

Four studies reported common clinical indicators of oral health, PI and GI. PI assesses the presence and thickness of plaque, while GI assesses gingiva swelling and bleeding. Both are rated on a scale from zero to three [63,64].

Purunaik and colleagues [52] recruited 90 participants to evaluate the benefits of the Darolac^®^ probiotic mouthwash on plaque and gingivitis. Children were randomized into three groups: probiotic, CHX, and placebo. After 14 days, PI scores were significantly reduced in the CHX and probiotic groups, but not in the placebo group. Similar results were observed for GI scores, with the difference that the probiotic was significantly more effective than the CHX mouthwash after 14 days of intervention.

The study of Thakkar et al. [51] used a similar design with 90 participants randomized into a probiotic, CHX or placebo group. After 14 days of intervention, PI scores were significantly lower in the probiotic and CHX groups compared to the placebo group. The difference between the probiotic and CHX groups was not statistically significant, suggesting that the two interventions were equivalent. However, at follow-up three weeks after the intervention, PI scores were significantly lower in the probiotic group compared to both the CHX and placebo groups, with no significant difference between the latter two groups. Authors concluded that the probiotic mouthwash was more effective at inhibiting plaque accumulation than other tested products. While Purunaik et al. [52] and Thakkar et al. [51] recruited adolescents aged 13 to 16, another study using a similar designed focused on younger children aged 6 to 9. Bande et al. [54] recruited 30 children randomized into a probiotic, CHX and placebo group. After 14 days of intervention, both PI and GI scores were significantly improved in the probiotic and CHX groups when compared to the placebo. For GI scores specifically, the probiotic mouthwash was significantly more efficient than CHX.

Lastly, one study [53] recruited participants with intellectual disabilities, a population that faces many challenges with respect to oral health practices. The study included 30 participants who were randomized to receive the Darolac^®^ probiotic mouthwash or herbal mouthwash. At three months, this study had the longest duration of intervention of all studies included in the present review. At the end of the intervention, both groups showed a significant reduction in PI and GI scores.

In summary, studies show that Darolac^®^ is effective at reducing the accumulation of plaque and improving gingival health in children. Furthermore, it is as effective as other commercially available products, including CHX. Some studies even obtained better results with Darolac^®^ than CHX.

### 3.2. Acceptability of Products

In studies included in this review, participants had to use the allocated mouthwash every day for a period ranging from 14 days to three months, and, in most cases, they had to use it twice daily. Issues with the acceptability of investigational products, such as finding that the mouthwash has an unpleasant taste, could compromise compliance with the study protocol and influence trial outcomes. One study included in this review assessed the taste acceptance of Darolac^®^, CHX and sodium fluoride mouthwash in children under 12 [55]. Overall, children rated the taste of their allocated mouthwash as “good”, with no difference between groups. For the duration of the intervention, children’s compliance with the study protocol was not affected by the taste of the investigational products.

### 3.3. Effects on Oral Pathogens

Two studies assessed the effect of Darolac^®^ against *S. mutans,* an oral pathogen whose counts are elevated in periodontal conditions [50,55]. Both studies used a 14-day intervention and measured outcomes at the end of the intervention. Jindal et al. [50] compared the Darolac^®^ probiotic mouthwash with a placebo and another probiotic solution, Sporlac^®^ (*Bacillus coagulans* SNZ 1969; Sanzyme Biologics Private Limited, Hyderabad, India). Both probiotics significantly reduced salivary *S. mutans* counts compared to the placebo, with no significant difference between both probiotics. Gedam and Katre [55] compared Darolac^®^ mouthwash to CHX and a sodium fluoride solution. Both CHX and Darolac^®^ significantly reduced the counts of salivary *S. mutans* after 14 days, with no significant differences between the two. In summary, Darolac^®^ was as effective as CHX and another commercially available probiotic solution to reduce counts of *S. mutans* over a period of 14 days.

## 4. Darolac^®^ for Adults

As it is related to a general decline in oral health, the prevalence of periodontal conditions increases with age. Adults may also hold long-term habits that put them more at risk of developing periodontal conditions, such as smoking or drug use. In addition, they may have diseases with serious consequences for oral health, such as head-and-neck cancer. The use of probiotics mouthwash has been investigated in both generally healthy individuals and participants with various health conditions.

### 4.1. Clinical Indicators of Oral Health

Four clinical trials measured PI and GI [57,58,60,61]. A recent study measured similar indicators of oral health that have been proposed over the past 15 years, the marginal plaque index (MPI) and bleeding on interdental brushing (BOIB) [62]. Briefly, MPI scores the presence (1) or absence (0) of plaque within eight equal areas of a tooth. BOIB consists of inserting a light brush interdentally and moving it along the surface of the gingiva. The presence or absence of bleeding is recorded across several interdental sites.

Jindal et al. [57] recruited participants with gingivitis aged 20 to 35. Participants were randomized into the placebo or the Darolac^®^ group. After 14 days of intervention, there was a significant difference in the mean changes of both PI and GI scores for the probiotic group, and significant differences in PI and GI scores between the probiotic and placebo groups. Using a similar design in a study of individuals with periodontitis, Tapashetti et al. [60] found that PI and GI scores were significantly reduced in the probiotic group after a 14-day intervention compared to baseline. Scores were also significantly reduced in the probiotic group compared to the placebo group. Ranjith et al. [61] also compared Darolac^®^ to a placebo in individuals with periodontitis but extended the duration of the intervention to 30 days and collected follow-up data at 90 days, that is, 60 days after the end of the intervention. In the probiotic and the placebo groups, both the PI and GI scores were significantly reduced at the end of the 30-day intervention compared to baseline. The GI score was significantly reduced in the probiotic compared to the placebo group, but there was no significant difference in PI scores between groups at the end of the intervention. At follow-up, a significant reduction in PI and GI scores was still observed in both groups compared to baseline, but again, only the GI score was significantly different between the placebo and probiotic group. Other indicators of periodontal health, such as the periodontal pocket depth (PPD) and the clinical attachment level (CAL), were also measured throughout the study. After 30 and 90 days, both PPD and CAL scores had improved in the probiotic and the placebo groups. Although group differences were not statistically significant, the probiotic group had higher PPD and CAL scores than the placebo group at baseline, indicating poorer oral health. At the end of the intervention, the placebo group had significantly lower scores compared to the probiotic group. However, after 90 days, the probiotic group had lower PPD and CAL scores than the placebo group, showing the continuity and maintenance of improvements.

Two studies compared the Darolac^®^ mouthwash to other products instead of a placebo. Deshmukh et al. [58] compared the effects of Darolac^®^, CHX, and herbal mouthwash on gingival health. Each study group included 15 participants, and the intervention lasted for 14 days. At the end of intervention, there was no significant difference in the efficacy of all three mouthwashes with regards to PI and GI scores, as well as to oral hygiene status. The study therefore shows that the Darolac^®^ probiotic formula, used as a mouthwash, can be an effective alternative to the CHX mouthwash currently used.

Lastly, the study of Boyapati et al. [62] randomized participants to receive either Darolac^®^, CHX, aloe vera or povidone–iodine mouthwash for a total of 28 days. At baseline, 14 and 28 days, both MPI and BOIB were measured. After 14 days of intervention, Darolac^®^ and povidone–iodine were more effective at reducing MPI and BOIB scores than the CHX mouthwash. Furthermore, both MPI and BOIB scores were significantly lower in the Darolac^®^ group compared to the povidone–iodine group. After 28 days, Darolac^®^ and povidone–iodine were still more effective at reducing MPI and BOIB scores than CHX. However, the difference between Darolac^®^ and povidone–iodine was only statistically significant for BOIB scores.

These studies show that Darolac^®^ is effective at reducing plaque accumulation and improving gingival health in generally healthy adults. Moreover, it is at least as effective, if not more so, than other commercially available products, including CHX and povidone–iodine.

### 4.2. Effect on Pathogens

Three studies assessed the activity of Darolac^®^ against oral pathogens [56,59,60]. Jothika et al. [56] aimed to compare the effects of different mouthwash solutions on *S. mutans* counts found in plaque samples. The study had four arms: placebo (distilled water), CHX, fluoride mouthwash and Darolac^®^. After completing oral prophylaxis, they collected plaque samples at baseline, and again 7, 14, and 30 days into the intervention. At all measurement points during the intervention, the probiotic group had the lowest mean CFU/mL of *S. mutans* count of all four groups. Furthermore, the three intervention groups (probiotic, CHX and fluoride) were significantly different from the placebo group, but all had similar counts of *S. mutans* after 30 days.

Another study assessed the effect of the probiotic formula on pathogenic strains found in subgingival plaque [60]. This study recruited adults with chronic periodontitis who were randomized into two groups: Probiotic (Darolac^®^) or placebo (distilled water). The pathogenic bacteria assessed in this study were *Porphyromonas gingivalis*, *Treponema denticola*, and *Tannerella forsythia,* also known as the red complex bacteria [30,31,32]. After 14 days of intervention, mean cell counts of red complex bacteria were significantly reduced compared to baseline in the probiotic group, but the change was not statistically significant in the placebo group. Intergroup analysis showed that *T. denticola* and *T. forsythia* bacterial counts were significantly reduced in the probiotic group when compared to the placebo group.

The study by Doppalapudi et al. [59] assessed the effect of the probiotic formula on the presence of pathogens from the *Candida* species. This study targeted a particular population of adults who underwent radiotherapy for head-and-neck cancer, as this therapy may impact the oral microbiome of patients. Participants were provided with the Darolac^®^ probiotic mouthwash, clotrimazole, or both, for an intervention lasting 30 days. At the end of the intervention, both groups who took the probiotic showed a significant reduction in salivary *Candida* spp. counts. In fact, both the probiotic alone and in combination with clotrimazole were significantly more effective at reducing *Candida* spp. counts than clotrimazole alone.

Lastly, although they did not directly measure the presence of pathogens, Ranjith et al. [61] collected indicators that can provide information about oral microbiota composition, such as salivary pH and secretory antibody levels (IgA). For instance, *S. mutans*, a pathogen involved in several oral health conditions, is both acidogenic and aciduric, meaning that it increases acidity (lowers pH) and has a good tolerance for acidic environments. Therefore, lower pH can be used as an indicator for the presence of *S. mutans* in the oral cavity [33,65]. Similarly, the presence of pathogens can trigger an immune response, which can be measured by tracking IgA levels [65]. In their study, Ranjith et al. [61] observed that salivary IgA levels were significantly increased after 30 days in the probiotic group, while values were similar to baseline at 90 days. When compared to the placebo group, IgA levels in the probiotic group were significantly higher at both timepoints. For salivary pH, there was a significant increase (lower acidity) for the probiotic group at 30 days followed by a decrease, but levels were still significantly higher than baseline at the 90-day follow-up. There was a statistically significant difference between groups at both timepoints. Authors concluded that a 30-day use of probiotics helped improve clinical parameters of oral health in participants with periodontitis.

In summary, Darolac^®^ can be used to curtail the proliferation of pathogens in the oral cavity. In studies where it was compared to other available oral health products, it was shown to be as effective [56] or even superior [59] in reducing counts of oral pathogens.

### 4.3. Special Considerations for Participants with Health Conditions

While most studies recruited participants with good overall health and no oral conditions, some studies recruited participants with periodontal conditions, which has implications for the indication of the intervention. In studies of generally healthy participants, probiotics are used as a complement to regular oral hygiene practices (toothbrushing, flossing) to maintain good oral health. However, in participants with periodontal conditions, probiotics are used to restore homeostasis in the face of dysbiosis. In that context, it is interesting to note that studies of participants with gingivitis [57] and periodontitis [60,61] found that the Darolac^®^ probiotic formulation was effective for improving oral health over an intervention period ranging 14 to 30 days, which is comparable to the duration of intervention used in studies of healthy participants. Furthermore, in two studies, benefits were maintained after the discontinuation of the intervention, which can suggest a return to homeostasis that can be maintained with regular oral hygiene practices. Lastly, in the study of Tapashetti et al. [60], improvements could be linked with reductions in counts of oral pathogens. The study of Ranjith et al. [61] also reported results related to acidity and immune response which were consistent with a reduction in oral pathogens. Overall, these results indicate that Darolac^®^ confers oral health benefits even when inflammation is present and has already led to the development of periodontal symptoms. Beneficial effects can be related to probiotics’ effects on oral pathogens and appear to contribute to the resolution of dysbiosis in a significant manner.

One reviewed study recruited individuals who had recently completed radiotherapy for head-and-neck cancer. As explained by Doppalapudi et al. [59], these individuals are at high risk of developing oral candidiasis due to salivary gland tissue damage that results in altered salivary flow and quality. Notably, hyposalivation has been related with the proliferation of *Candida albicans* and incidence of candidiasis. In their study, both participants who took the probiotic and clotrimazole and participants who took the probiotic alone saw decreased counts of *Candida* spp. to similar levels. While antifungal agents (e.g., clotrimazole, fluconazole) are effective against candidiasis, they are associated with adverse effects such as loss of appetite, nausea, and diarrhea. Reducing adverse effects and contributing to better quality of life represents an important goal to support individuals who have just completed radiotherapy, and this study supports the use of Darolac^®^ as an effective solution to curtail the overgrowth of bacteria from the *Candida* species in the oral cavity.

## 5. Discussion

The clinical studies included in this review demonstrate that the Darolac^®^ probiotic formula is an appropriate support for oral health in generally healthy individuals and in individuals with gingivitis and periodontitis. Here, we described 13 clinical trials that compared Darolac^®^, a probiotic combination of *Lactobacillus acidophilus* Rosell^®^-52, *Lactobacillus rhamnosus* Rosell^®^-11, *Bifidobacterium longum* Rosell^®^-175 and *Saccharomyces boulardii* CNCM I-1079 with CHX mouthwash [51,52,54,55,56,58,62] and/or other alternative mouthwashes used to support oral health [50,53,56,58,59,62]. Overall, Darolac^®^ was at least as effective as the CHX mouthwash to improve several parameters of oral health, such as plaque accumulation, gingival health, and pathogenic bacterial growth. When compared with other alternative solutions such as herbal mouthwashes, fluoride, clotrimazole or povidone-iodine, Darolac^®^ also induced comparable or significantly better oral health outcomes. Lastly, for the studies comparing Darolac^®^ exclusively to a placebo mouthwash, all showed significant improvement of clinical oral health parameters after the intervention [57,60,61].

Darolac^®^ includes probiotics with established safety. The safety of the Darolac^®^ formulation was further supported by its use in both children and adults in the studies included in the present review. While monitoring of adverse events was not explicitly described in all studies, four studies monitored for adverse events, and none were recorded. Therefore, Darolac^®^ is safe to use by children (from 6 to 16 years) and adults (from 18 years) for the maintenance and improvement of oral health.

If not managed properly, gingivitis can progress to periodontitis and lead to pain, loss of tooth attachment, and swelling of gums that pull away from the teeth, creating pockets where bacteria can grow and further exacerbate symptoms [19]. While these consequences are serious and debilitating, periodontitis has also been associated with several diseases and conditions, including diabetes, rheumatoid arthritis, chronic kidney disease, and cardiovascular diseases [66,67]. Pro-inflammatory responses from oral pathogens, notably from the *Streptococcus* genus, which result in an increase in C-reactive protein levels and oxidative stress, have been identified as a key component of the mechanisms that underlie this association [66,67]. Furthermore, decreased abundance of nitrate-reducing bacteria, which limits the bioavailability of nitric oxide, has been associated with some metabolic and cardiovascular diseases [39,40]. A consensus report produced jointly by the European Federation of Periodontology and the American Academy of Periodontology has identified periodontitis as a non-traditional modifiable risk factor for cardiovascular disease [67], which clearly highlights the importance of preventing and treating periodontal conditions [66]. The use of effective adjuvants that can act directly on inflammation through the restoration of homeostasis in the oral cavity, such as the Darolac^®^ probiotic formulation, appears particularly indicated in this context.

One of the strengths of the current review is the inclusion of studies that all used the Darolac^®^ probiotic formulation and compared it to a placebo or other commonly used mouthwashes, including CHX, which is the standard adjunctive solution for periodontal conditions. Taken together, the studies covered a wide age range and included participants with generally good health, but also participants with periodontal conditions. Participants were from different regions in India, which ensures a certain homogeneity of results while also accounting for regional differences in practices and diets. Lastly, several studies used common clinical indicators of oral health, which facilitates their comparison.

This review also has some limitations. Participants were asked to use the probiotic or comparator for 14 days in eight studies [50,51,52,54,55,57,58,60], 28 or 30 days in four studies [56,59,61,62] and three months (90 days) in one study [53]. While the duration of intervention was short in most studies, it was sufficient to generate positive effects. Still, it is possible that some benefits might emerge or be strengthened after longer use of the probiotic formulation. Furthermore, only two studies collected follow-up measures one week [51] and two months [61] after discontinuation of the intervention, respectively. Studies with a longer duration of intervention and a longer follow-up period would help describe the long-term effects of continued probiotic consumption and the maintenance of effects after discontinuation of intervention. Only a few studies monitored the presence of oral pathogens over the duration of the intervention, and the specific pathogens monitored in each study varied. More systematic reporting of those results across studies would allow more in-depth discussion of the effect of probiotics on oral pathogens. Further characterization of this probiotic formulation’s antimicrobial activity represents the next step in elucidating its mechanism of action. No study reported results on oral cavity microbiota composition, but one study reported indicators that can, albeit indirectly, indicate the presence of bacteria that modulate acidity and immune response. Since probiotics have the potential to influence microbial flora and exert several effects with overall positive impacts on oral health, future studies should report microbiota composition before and after the intervention and report more specifically on the presence of bacteria known to induce lower acidity and immune responses against pathogens, and bacteria with key metabolic activity. Furthermore, while the oral microbiota of people with good oral health and periodontal conditions has been characterized using 16S sequencing and state-of-the-art technological advances (metagenomics, transcriptomics, proteomics, metabolomics) [20,68], only a handful of clinical trials have used high-throughput molecular methods to assess the effects of probiotics on oral microbiota before and after an intervention [69]. Widespread and consistent use of those methods would contribute to a more rigorous measure of outcomes and would provide invaluable information on probiotics’ mechanisms of action for the maintenance and restoration of oral health. Lastly, inflammation is a key factor in the development and aggravation of periodontal conditions, but no study measured biomarkers of inflammation across the duration of intervention and at follow-up. While inflammation was assessed clinically through the collection of indicators of gingival swelling and bleeding (GI, BOIB), it was not directly quantified through the collection of biomarkers (e.g., cytokines). This seems like an important gap in the literature, considering that reducing inflammation is a key target in the management of periodontal conditions.

## 6. Conclusions

This review provides a summary of clinical trials using the Darolac^®^ probiotic formulation for the maintenance and improvement of oral health in children and adults. Overall, studies show that Darolac^®^ is a safe and natural alternative to conventional mouthwash. It is an effective adjuvant to oral health in generally healthy individuals and people with periodontal conditions, with effects comparable to the product most commonly used as an adjunctive solution, chlorhexidine mouthwash. More studies are needed to understand the effects of the Darolac^®^ probiotic formulation on oral microbiota composition and on its promotion of anti-inflammatory factors that help manage periodontal conditions.

## Figures and Tables

**Table 1:** Characteristics of reviewed studies.

First Author, Year, and Reference	Study Design	*N,* Population, and Age Range	Study Arms, *n*	Darolac^®^ Regimen	Intervention Duration	Baseline Visit	Clinical Parameters	Assessment Timepoints	Adverse Events	Main Findings
Studies conducted with children										
Jindal, 2011 [50]	DB-RCT	150 Children 7–14 years	Placebo, *n* = 50 Darolac^®^, *n* = 50 Sporlac^®^, *n* = 50	1 sachet of Darolac^®^ 1.25B (mixed in 20 mL of water) daily	14 days	Measures taken Instructions given	*Streptococcus mutans* CFU/mL of saliva	14 days	n.r.	After 14 days, *S. mutans* counts were reduced significantly in children from both probiotic groups when compared to the placebo group.
Thakkar, 2013 [51]	DB-RCT	90 Children 13–15 years	Placebo, *n* = 30 Darolac^®^, *n* = 30 Chlorhexidine 0.12%, *n* = 30	1 sachet of Darolac^®^ 1.25B (mixed in 10 mL of water) daily	14 days	Measures taken Oral prophylaxis Instructions given	PI	14 days, 3 weeks	n.r.	After 14 days and at the 3-week follow-up, the probiotic and Chlorhexidine groups had significantly lower PI scores than the placebo group, but there was no significant difference between those two groups.
Purunaik, 2014 [52]	DB-RCT	90 Children 15–16 years	Placebo, *n* = 30 Darolac^®^, *n* = 30 Chlorhexidine 0.2%, *n* = 30	1 sachet of Darolac^®^ 1.25B (mixed in 20 mL of water) twice daily	14 days	Measures taken Instructions given	PI GI	14 days	n.r.	After 14 days of intervention, PI and GI scores were significantly lower in the probiotic group compared to the placebo and Chlorhexidine groups.
Aarthy, 2021 [53]	RCT	30 Children with ID 12–15 years	Darolac^®^, *n* = 15 HiOra, *n* = 15	1 sachet of Darolac^®^ 1.25B (mixed in 20 mL of water) twice daily	3 months	Measures taken Instructions given Provided toothbrush and toothpaste	PI GI	3 months	n.r.	After 3 months, both intervention groups had a significant reduction in PI and GI scores.
Bande, 2021 [54]	DB-RCT	30 Children 6–8 years	Placebo, *n* = 10 Darolac^®^, *n* = 10 Chlorhexidine, *n* = 10	1 sachet of Darolac^®^ 2.5B (mixed in 20 mL of water) daily, 5 mL at a time	14 days	Measures taken Oral prophylaxis Instructions given	PI GI	14 days	n.r.	At 14 days, both the probiotic and the Chlorhexidine group had significantly lower PI scores than the placebo group, but the difference between those groups was not significant. The probiotic group had significantly lower GI scores than both the placebo and Chlorhexidine groups.
Gedam, 2022 [55]	TB-C-RT	51 Children 8–12 years	Initial allocation (crossover design): Placebo, *n* = 16 Darolac^®^, *n* = 19 Chlorhexidine 0.12%, *n* = 16	1 sachet of Darolac^®^ 1.25B (mixed in 10 mL of water) twice daily	14 days	Measures taken Instructions given: Maintain normal hygiene practices with non-fluoridated toothpaste	*Streptococcus mutans* CFU/mL of saliva Taste acceptance	14 days	No AEs	Darolac^®^ and Chlorhexidine led to a significant reduction in *S. mutans* counts after 14 days. There was no significant difference between interventions.
Studies conducted with adults										
Jothika, 2015 [56]	SB-RCT	52 Adults 18–25 years	Placebo, *n* = 13 Darolac^®^, *n* = 13 Chlorhexidine 0.2%, *n* = 13 Fluoride, *n* = 13	1 sachet of Darolac^®^ (mixed in 10 mL of water) twice daily	30 days	Measures taken Oral prophylaxis and teaching of modified Bass technique Instructions given	*Streptococcus mutans* CFU/mL of plaque sample	7, 14 and 30 days	n.r.	After 30 days of intervention, all three intervention groups had lower *S. mutans* counts compared to the placebo. There was no significant difference between interventions.
Jindal, 2017 [57]	DB-RCT	30 Individuals with gingivitis 20–35 years	Placebo, *n* = 15 Darolac^®^, *n* = 15	1 sachet of Darolac^®^ 1.25B (mixed in 20 mL of water) twice daily	14 days	Measures taken Instructions given	PI GI	14 days	n.r.	Change in PI and GI scores were significant between the baseline and post-intervention measure and between the probiotic and placebo groups.
Deshmuk, 2017 [58]	TB-RCT	45 Adults 18–21 years	Chlorhexidine 0.2%, *n* = 15 Darolac^®^, *n* = 15 HiOra, *n* = 15	1 sachet of Darolac^®^ 1.25B (mixed in 20 mL of water) twice daily	14 days	Measures taken Oral prophylaxis Instructions given Provided toothbrush and toothpaste	OHI-S PI GI	7 and 14 days	n.r.	There was no difference between groups in PI scores, GI scores, and OHI-S after 14 days of intervention.
Doppalapudi, 2020 [59]	RCT ^a^	90 Adults who underwent radiotherapy for head-and-neck cancer 24–80 years	Darolac^®^, *n* = 30 Clotrimazole 1%, *n* = 30 Darolac^®^ + Clotrimazole 1%, *n* = 30	1 sachet of Darolac^®^ 1.25B (mixed in a liquid) thrice daily	30 days	Measures taken Instructions given	*Candida* CFU/mL of saliva	30 days	No AEs	Both probiotic groups showed a significantly lower count of *Candida* spp. in saliva samples after 30 days of intervention. The difference between probiotic groups was not significant.
Tapashetti, 2022 [60]	DB-RCT	20 Individuals with chronic periodontitis 18–55 years	Placebo, *n* = 10 Darolac^®^, *n* = 10	1 sachet of Darolac^®^ 1.25B (mixed in 20 mL of water) twice daily	14 days	Measures taken Oral prophylaxis Instructions given	PI GI Microbes detection in subgingival plaque samples	14 days	n.r.	In the probiotic group, GI and PI scores were significantly lower after 14 days compared to baseline. Scores at 14 days were lower in the probiotic group compared to the placebo group. Microbial counts were significantly lower after 14 days than at baseline in the probiotic group. Microbial counts were significantly lower in the probiotic group compared to the placebo group.
Ranjith, 2022 [61]	TB-RCT	60 Individuals with stage II periodontitis over 30	Placebo, *n* = 30 Darolac^®^, *n* = 30	1 sachet of Darolac^®^ 1.25B (mixed in 20 mL of water) twice daily	30 days	Measures taken Oral prophylaxis Instructions given	PI GI PPD CAL Periodontal status Salivary pH and IgA	30 and 90 days	No AEs	30 days: GI, PI, PPD and CAL were significantly lower than at baseline in both groups. GI was significantly lower in the probiotic group compared to the placebo group, while PPD and CAL were significantly lower in the placebo group compared to the probiotic group. IgA and pH levels were increased in the probiotic group vs. baseline and placebo group. At 90 days: GI, PI, PPD and CAL were significantly lower than at baseline in both groups. GI, PPD and CAL were significantly lower in the probiotic group compared to the placebo group. In the probiotic group, IgA levels were comparable to baseline but increased compared to the placebo group. pH levels were increased in the probiotic group vs. baseline and the placebo group.
Boyapati, 2024 [62]	RCT	60 Adults 18–45 years	Darolac^®^, *n* = 15 Chlorhexidine, *n* = 15 Aloe vera, *n* = 15 Povidone-iodine, *n* = 15	Use twice daily	28 days	Measures taken Oral prophylaxis Instructions given Provided toothbrush and toothpaste	MPI BOIB	14 and 28 days	No AEs	All groups showed a decrease in MPI and BOIB scores. The decrease in MPI and BOIB in the probiotic group was comparable to the one observed in the Chlorhexidine group.

Legend. RCT: Randomized Controlled Trial; SB: Single-blind; DB: Double-blind; TB: Triple-blind; n.r.: not recorded; PI: plaque index; GI: gingival index; OHI-S: oral hygiene index simplified; PPD: periodontal pocket depth; CAL: Clinical attachment level; MPI: marginal plaque index; BOIB: bleeding on interdental brushing. ^a^ Participants were not blinded as the Darolac^®^ and Clotrimazole regimens are not administered in the same way.

## Data Availability

No new data were created or analyzed in this study.
